# Magneto-actuated cell apoptosis by biaxial pulsed magnetic field

**DOI:** 10.1038/s41598-017-11279-w

**Published:** 2017-09-07

**Authors:** De Wei Wong, Wei Liang Gan, Ning Liu, Wen Siang Lew

**Affiliations:** School of Physical and Mathematical Sciences, Nanyang Technological University, 21 Nanyang Link, Singapore, 637371 Singapore

## Abstract

We report on a highly efficient magneto-actuated cancer cell apoptosis method using a biaxial pulsed magnetic field configuration, which maximizes the induced magnetic torque. The light transmissivity dynamics show that the biaxial magnetic field configuration can actuate the magnetic nanoparticles with higher responsiveness over a wide range of frequencies as compared to uniaxial field configurations. Its efficacy was demonstrated in *in vitro* cell destruction experiments with a greater reduction in cell viability. Magnetic nanoparticles with high aspect ratios were also found to form a triple vortex magnetization at remanence which increases its low field susceptibility. This translates to a larger magneto-mechanical actuated force at low fields and 12% higher efficacy in cell death as compared to low aspect ratio nanoparticles.

## Introduction

Magnetic nanoparticles (MNPs) have been intensively researched for biomedical applications, such as magnetic resonance imaging, magnetic hyperthermia and drug delivery, to overcome the shortcomings of current diagnosis and therapy methods^1–4^. Compared to other nanomaterials, MNPs can be remotely controlled by externally applied magnetic fields. The resulting magnetic response, in the form of mechanical force or heat generation, is governed by magnetic field configuration, magnetic moment and magnetic susceptibility of the MNPs, all of which offer exciting possibilities in their manipulation^5^. Magnetic hyperthermia uses high frequency alternating magnetic fields in the kHz‒MHz range that induce a localized heating that allows targeted cell death at a critical temperature of above 42 °C^3, 6, 7^. However, its invasiveness and high difficulty in targeting a specific cancerous area with homogenous heat generation poses a fundamental problem, in addition to the requirement of high frequency and strong magnetic fields^8, 9^.

In recent years, it has been demonstrated that the mechanical force exerted by microdiscs under an alternating magnetic field of less than 100 Hz is sufficient to achieve cell apoptosis^10–12^. In contrast to achieving tumor regression through the effects of hyperthermia, the magneto-mechanical cell destruction method provides a non-invasive and targeted treatment method with high specificity and efficiency. Magneto-mechanical actuation is achieved by the spatial rotation of the particles to align to the applied magnetic field by Brown relaxation, which produces a magnetic torque^13, 14^. Most experimental work uses superparamagnetic particles, with zero remanent magnetization and low saturation magnetization values, to avoid agglomeration occurring due to magnetic stray fields. Therefore, the use of soft magnetic materials, with closed spin-vortex magnetizations and high magnetization of saturation, eliminates the need of high magnetic fields for manipulation of these particles. Although the efficacy of relatively large microdiscs with a single vortex state is well understood^10–12^, there is little research work on multi-vortex MNP *in vitro* magneto-actuated cancer cell apoptosis effects.

In this study, we demonstrate the use of a biaxial pulsed magnetic field to achieve *in vitro* magneto-actuated cell apoptosis. Its effectiveness was demonstrated in the light transmissivity dynamics of MNPs and in the decrease in the viability of HeLa cells. Micromagnetic simulations show that the MNPs, with different aspect ratios, can form triple vortex states that increases the magnetic susceptibility and torque experienced by the MNPs. These results were further validated by their efficacy in inducing programmed cancer cell death.

## Results and Discussion

### Light Transmissivity Dynamics

Under an applied magnetic field, the MNPs will experience a torque that aligns its net magnetic moments to the applied field. This magnetic torque can be experimentally observed through the light transmissivity measurements of a suspension of MNPs, with diameter (*d*) of 150 nm and length (*l*) of 500 nm. In this experiment, a quadrupole electromagnet is employed to apply an external magnetic field in two orthogonal directions. A laser and photodiode measure the light intensity transmitted through a suspension of MNPs in deionized water (DI) under different applied magnetic field configurations. The viscosities and refraction indices of DI water and DMEM + 10% FBS are 0.88 cP and 0.94 cP, 1.33 and 1.345, respectively^15^. Both values are comparable and show consistent light transmissivity results. In uniaxial AC and DC pulsed magnetic field configuration, the magnetic field of 10 Oe is applied in a single direction using two opposite coils with AC or DC pulses. It is important to note that such a small amplitude magnetic field is insufficient in changing the magnetization states of MNPs, but enough to control the orientations of the MNPs. While in biaxial pulsed magnetic field configuration, the magnetic field is pulsed in two orthogonal directions. The MNPs are initially magnetized and saturated by applying a large magnetic field of 1 kOe. *I*_*max*_ corresponds to the maximum light intensity measured, while *I*_*min*_ is the minimum light intensity measured due to the misalignment of the MNPs caused by Brownian motion^10, 16^. The change in transmitted light intensity (*∆I* = *I*_*max*_ − *I*_*min*_) is measured over a range of frequencies. The frequency response of *∆I* is representative of the MNP responsiveness to the applied magnetic field, which in turn is determined by the torque experienced by the MNP, and subsequently the resultant mechanical force exerted on the cells.

**Figure 1:**
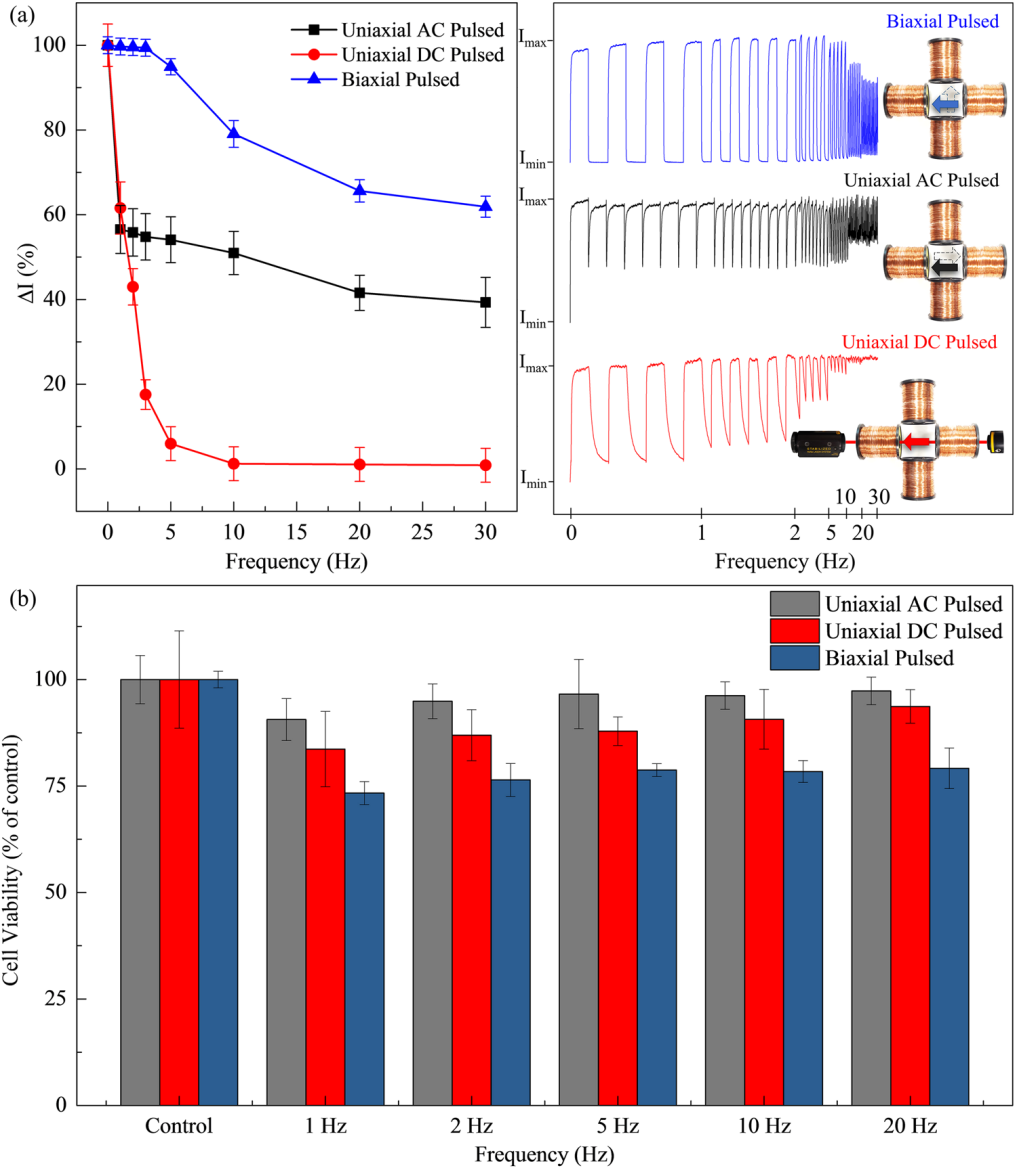
(**a**) The change in transmitted light intensity *∆I* measured over a range of frequencies under the different applied magnetic field configurations; uniaxial DC pulsed (red), uniaxial AC pulsed (black) and biaxial pulsed magnetic fields (blue). The right inset shows the change in amplitude for increasing frequencies (1–30 Hz) and experimental setup with corresponding applied field directions. (**b**) Cell viability of HeLa cells after magneto-actuated cell apoptosis treatment for different applied magnetic field configurations.

Magneto-actuated cell apoptosis relies on two mechanisms to induce a force on the cell walls. The first of which is the Brown relaxation which describes the rotation of the MNPs to the direction of the applied field due to an induced magnetic torque. The Brown relaxation time *t*_*B*_ characterizes the speed of rotation and was found to be 24 ms in our experiment by measuring the time taken for the light intensity to reach 0.9*I*_*max*_ from 0.1*I*_*max*_. The second mechanism is the random rotational Brownian motion which introduces disorder in the alignment MNPs after the magnetic field is removed^13^. However, this rotation is slower, with the rotational Brownian motion time *t*_*R*_ measured to be 400 ms. In uniaxial field configurations, the frequency response is limited by the large *t*_*R*_ as shown in Fig. 1(a), where the intensity oscillation amplitude of the uniaxial AC and DC pulsed magnetic fields shows a large decrease with increasing frequency. In contrast, the biaxial pulsed field shows a high *∆I* of 94.9–99.7% at low frequencies of 1–5 Hz. The application of magnetic field in orthogonal directions has two advantages. Firstly, it is no longer limited by the large *t*_*R*_ as the need for MNP misalignment due to the random rotational Brownian motion is eliminated^17^. This results in the diminishing value of *∆I* being less substantial at higher frequencies. Secondly, a biaxial field configuration also ensures that the magnetic field is applied at a larger angle to the MNPs, hence maximizing the magnetic torque. The light transmissivity dynamics demonstrates that the biaxial field configuration can solicit a faster and stronger magnetic response from the MNPs across a wide range of frequencies.

### Magneto-Actuated Cell Apoptosis

To investigate the efficiency of the various magnetic field configurations, a magnetic field of 140 ± 10 Oe was applied to the suspension of HeLa cells with a concentration of 0.1 mg/ml of MNPs, for a range of frequencies between 1‒20 Hz over a period of 10 mins each. During the experiments, the temperature of cell medium was monitored by an infrared thermometer and kept at 23.0 ± 0.5 °C, ensuring the contribution of magnetic hyperthermia is negligible. From Fig. 1(b), the cell viability after the application of uniaxial AC pulsed magnetic field is at above 90% for all frequencies, which indicates low cell death induced by magneto-mechanical effects. This is reflective of the light transmissivity results in Fig. 1(a), where the MNP is unable completely rotate to align to the field direction, resulting in a smaller magnetic torque experienced by the MNP, and hence weaker mechanical force exerted on the cells. The cell viability also shows an increase in average effectiveness of 6.6% for uniaxial AC to DC pulsed magnetic field. However, at frequencies above 10 Hz, the DC pulsed magnetic field becomes increasingly ineffective in rotating the MNPs by Brown relaxation (*∆I* ≈ 0%), which resulted in falling toxicity, as shown in Fig. 1(b).

The effects of magneto-actuated cell apoptosis are greatly increased by applying a biaxial pulsed magnetic field. Although a steady decline in the effectiveness of cell destruction with increasing frequencies was observed, the effects of magneto-actuated induced cell death is still apparent at high frequencies. The lower responsiveness of MNPs to the magnetic field demonstrates that a smaller magnetic torque is generated. Therefore, reinforcing that lower frequencies are more effective for magneto-actuated cell apoptosis, with the biaxial pulsed magnetic field showing an increase in average efficacy of 17.9% and 11.3% over the uniaxial AC and DC pulsed magnetic field, respectively.

**Figure 2:**
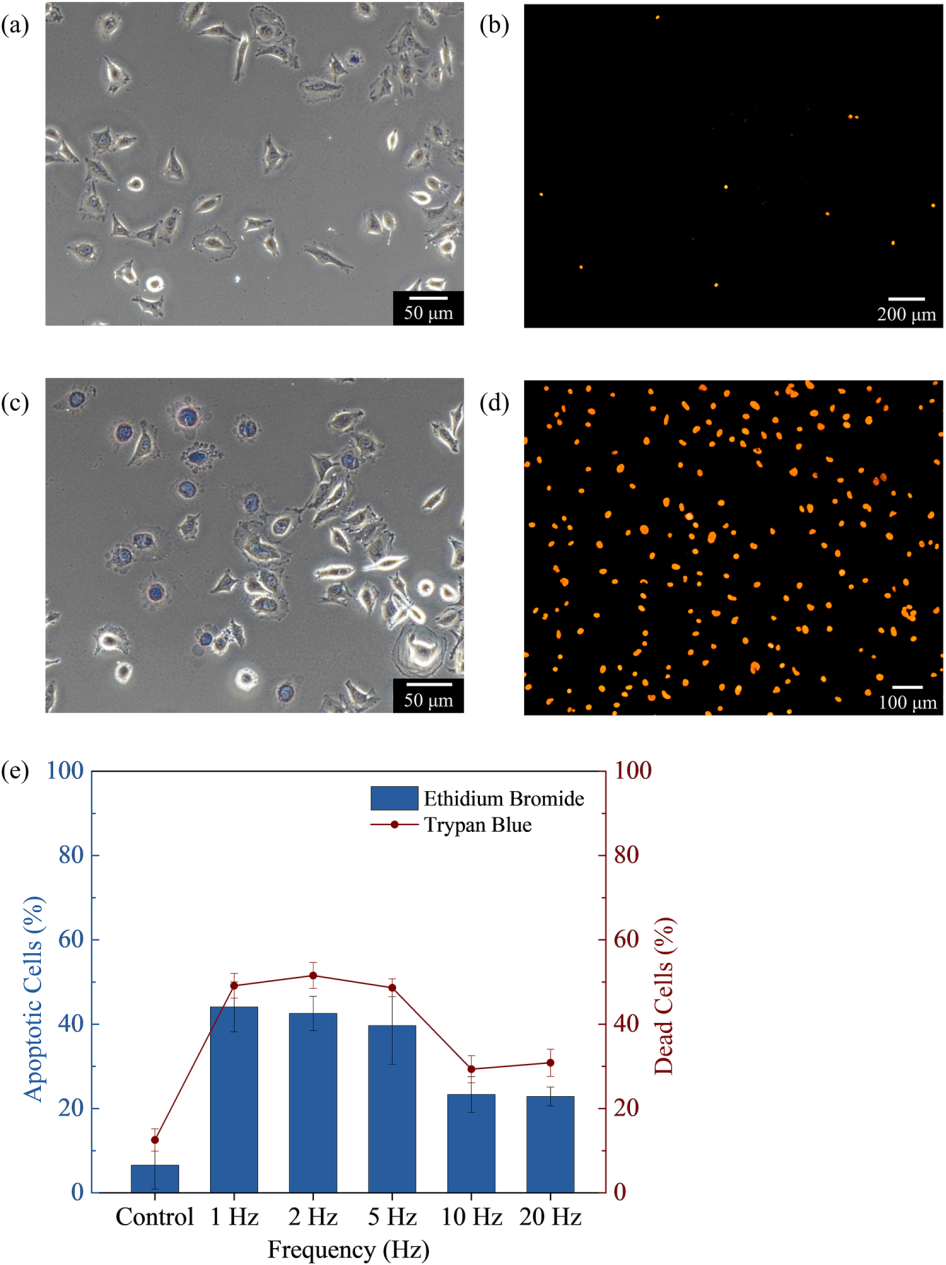
*In vitro* cell apoptosis by biaxial pulsed magnetic field. Optical image of HeLa cells and NiFe MNPs (**a**,**b**) no field control and (**c**,**d**) with magnetic field treatment. The cells (**a**,**c**) were stained with Trypan blue (TB) to obtain the dead cells count and (**b**,**d**) stained with Ethidium Bromide (EB) to observe apoptotic changes. (**e**) The quantified data from TB-stained and EB-positive fluorescent cells for frequencies between 1‒20 Hz. The error bars denote the standard deviation across four wells.

Ferromagnetic microdiscs have been shown to activate mechanosensitive ion channels mediated by mechanical stimuli, which is able to initiate cell apoptosis and programmed cell death^10^. The activation of mechanosensitive channels, due to cell membrane stretching, leads to the increase of intracellular calcium^18, 19^. This prolonged exposure to high concentration of intracellular calcium triggers cell apoptosis^20–22^. After the application of the biaxial pulsed magnetic field, the cell death was analyzed by light and fluorescence microscopy. Optical images show drastic changes in the morphology of the HeLa cells structures. The control cells maintain an elongated cell structure, whereas the cells exposed to the applied magnetic field showed a more circular and rounded-off cell structure, as shown in Fig. 2(a) and (c), respectively. This is an apparent cell fractionation and redistribution of nucleic material^10^. These observed effects are characteristic of apoptosis via genomic DNA fragmentation. The percentage of dead cells was obtained by using haemocytometry and Trypan Blue (TB) solution. TB dye exclusion test is routinely used as a cell stain to assess cell viability, where only the non-viable cells are permeable to the dye.

To observe the apoptotic effects by fluorescence microscopy, the HeLa cells were stained with Ethidium Bromide (EB), which is a common dye used for DNA and RNA detection. EB enters only non-viable cells with damaged membrane and fluoresce with an orange colour, after binding to DNA^23, 24^. In the absence of an applied magnetic field, the control cells with MNPs have shown to be non-cytotoxic, as revealed in Fig. 2(b). While the HeLa cells after the magneto-actuated treatment exhibit signs of early apoptosis as indicated by bright nuclear staining with EB, as shown in Fig. 2(d). The quantified data from TB-stained and EB-positive fluorescent cells for frequencies between 1‒20 Hz are presented in Fig. 2(e). A larger number of apoptotic cells and dead cells were observed at low frequencies of 1–5 Hz. At higher frequencies, the number of apoptotic cells and dead cells decreased, but still showed signs of apoptosis. The TB staining of 49.1% at 1 Hz (12.6% for control) indicates that the MNPs in the presence of the biaxial magnetic field can induce sufficient mechanical force to compromise the integrity of the cell membrane. It was observed that some cells had change in morphology without EB staining, which can be attributed to necrotic cell death caused by a strong mechanical disruption to the cell structure^11^. Hence, Fig. 2(e) shows an overall higher percentage of dead cells count from the TB dye test as compared to the percentage of apoptotic cells.

### Comparison of Magnetic Nanoparticles with Different Aspect Ratios

**Figure 3:**
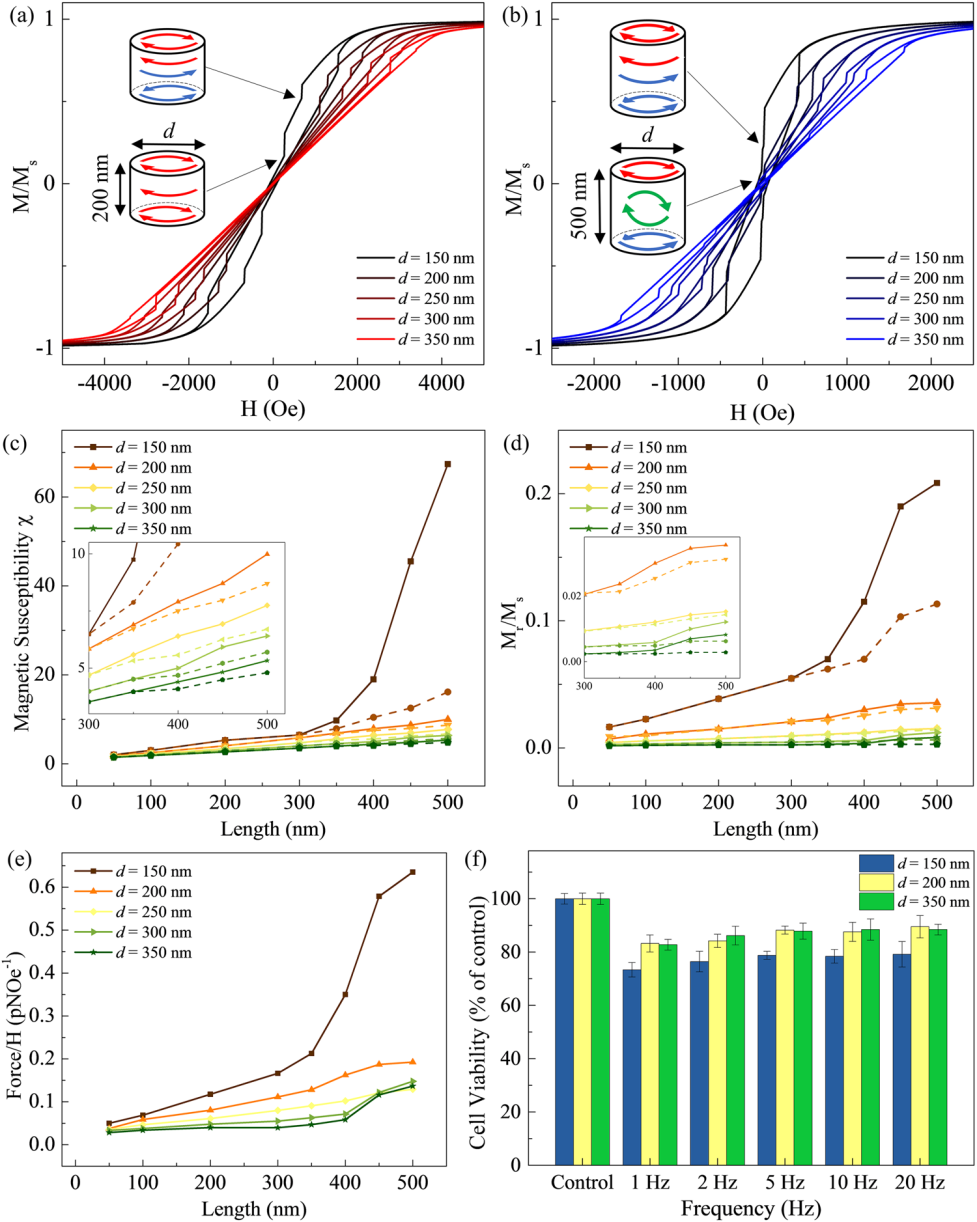
(**a**,**b**) The hysteresis loop of MNPs with *d* = 150–350 nm and *l* = 200 nm and 500 nm, respectively. (**c**,**d**) The low field magnetic susceptibility (*χ*) and magnetization M_r_/M_s_ was obtained as a function of MNP *d* = 150–350 nm and *l* = 50–500 nm for both single vortex state (dashed lines) and triple vortex state (solid lines). (**e**) The force exerted by MNPs with respect to applied magnetic field for *d* = 150–350 nm and *l* = 50–500 nm. (**f**) The comparison of cell viability of HeLa cells after magneto-mechanical cell destruction treatment using MNPs with *d* = 150–350 nm and *l* = 500 nm.

The hysteresis loops provide an insight on the magnetization states of the MNPs and its reversal mechanism under an applied magnetic field. Initially, the MNP was saturated by applying a large magnetic field along the MNP long axis and then relaxed. The simulations show that the MNPs have two distinct reversal processes with respect to an out-of-plane field depending on their length (*l*). At lengths *l* < 100 nm, only a single vortex nucleation is observed, characterized by a curling magnetization that is found in single domain circular nano-magnets^25, 26^. The interaction between the magnetostatic energy and exchange energy, favors an in-plane, closed flux domain structure which is the single vortex state. For lengths 100 < *l* < 300 nm, a double vortex nucleation occurs, consisting of a pair of anti-clockwise and clockwise vortex cores at the opposite ends of the MNPs. However, the annihilation of one vortex occurs as the magnetic field decreases, resulting in a single vortex state. The hysteresis loop for a MNP with *l* = 200 nm and *d* = 150–350 nm is shown in Fig. 3(a). As *l* increases to more than 300 nm, the annihilation of vortex at low fields is absent, whereas a third vortex core is nucleated on the curved surface of the MNP. In Fig. 3(b), the hysteresis loop for a MNP, with *l* = 500 nm and *d* = 150–350 nm, illustrates the reversal process from double vortex to triple vortex state.

By examining the hysteresis loops, the low field magnetic susceptibility (*χ*) was obtained as a function of MNP *l* and *d* for both single vortex state (dashed lines) and triple vortex state (solid lines), and tabulated in Fig. 3(c). The triple vortex state is a frustrated state that is found to be stable in ferromagnetic MNPs over a wide range of material parameters^16^. To obtain the single vortex state in MNPs with *l* > 300 nm, a clockwise bias field is initially applied to favor the formation of a single vortex magnetization in the MNPs. An increase in low field susceptibility as the magnetization state of the MNP switches from a single to triple vortex state at *l* > 300 nm is observed. Similarly, the remanent magnetization of the MNP is higher for the triple vortex state as compared to the single vortex state, as tabulated in Fig. 3(d). This increase is most significant for 150-nm-diameter MNPs, in contrast to ferromagnetic discs where a decrease in diameter or increase in length leads to a reduction in susceptibility^27, 28^. The magnetic torque exerted on a MNP by the applied magnetic field is given by, *τ* = *m* × *μ*_0_ *H* = *μ*_0_ *M*_*r*_ *VHsinθ*, where *M*_*r*_ is the magnetic moment of the MNP at remanence, *H* is the applied field, *μ*_0_ is the magnetic permeability (*µ*_0_ = 4π × 10^−7^ Hm^−1^), and *V* is the volume of the MNP $(V=\pi {(\frac{d}{2})}^{2}l)$. The angle *θ* is the angle between the applied magnetic field and plane of the MNP, which gives maximum torque when the field is at 90°. The force acting on the HeLa cells can be approximated by calculating the force exerted at the edge of the MNP, $F=\frac{2\tau }{l}$. The *F/H* (pNOe^−1^) graph shows that a 150-nm-diameter MNP is able to induce a significantly greater force as compared to the other MNPs, as shown in Fig. 3(e). At 140 Oe, the force for *d* = 150 nm, 250 nm and 350 nm MNPs are obtained to be 88.9 pN, 27.0 pN and 19.1 pN, respectively. Although a force of ~100 pN is needed to rupture the cell membrane physically^29–31^, only 0.5 pN is needed to activate ion channels leading to cell apoptosis^32, 33^.

Next, the effects of magneto-actuated cell apoptosis are compared for *d* = 150 nm, 250 nm and 350 nm MNPs (*l* = 500 nm), with triple vortex states, under the biaxial pulsed magnetic field. Similar trends of decreasing effectiveness at higher frequencies, with highest cell death recorded at 1 Hz, as shown in Fig. 3(f). A drop of 12% effectiveness in cell viability is also observed at all frequencies for both *d* = 200 nm and 350 nm MNPs as compared to *d* = 150 nm MNPs. This result is supported by simulations which showed that 150-nm-diameter MNPs are able to exert a force that is more than four times larger than MNPs with *d* > 200 nm.

## Conclusion

In summary, it has been demonstrated that a larger magnetic torque can be actuated on the MNP with a biaxial pulsed magnetic field that resulted in an increase *in vitro* magneto-actuated cell apoptosis. This is attributed to the orthogonal fields that eliminates the reliance on rotational Brownian motion. The light and fluorescence microscopy have shown the integrity of the cell membrane being compromised and the initiation of programmed cell death via apoptosis. Comparing MNPs of different aspect ratios, it was found that the 150-nm-diameter MNPs possessed the highest low field susceptibility and remanent magnetization, thus allowing a larger force to be exerted on the cells. This was seen in the cell viability test where the 150-nm-diameter MNPs produced 12% higher cell death.

## Methods

### Fabrication and characterization of magnetic nanoparticles

**Figure 4:**
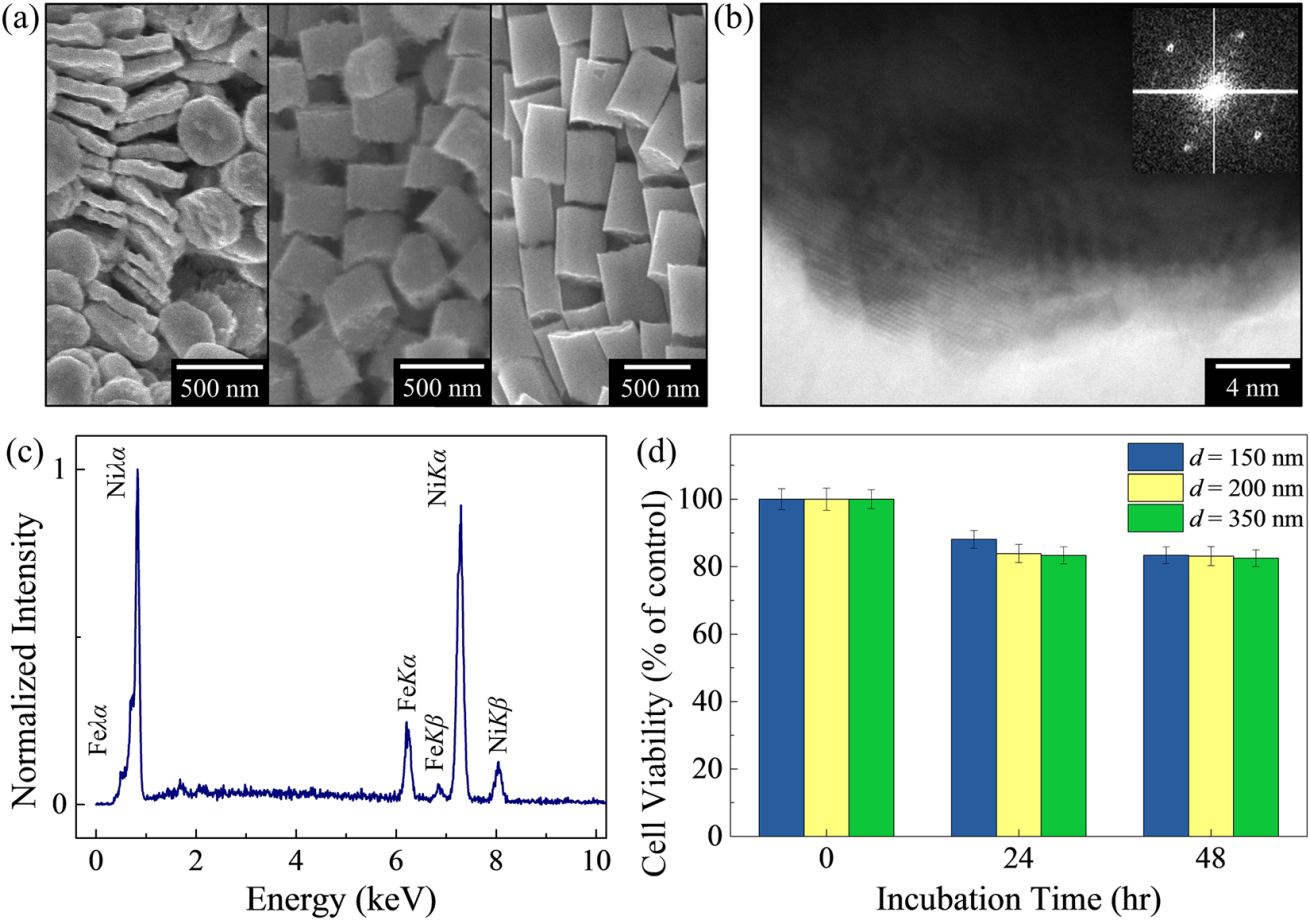
(**a**) SEM image of NiFe MNPs fabricated via template-assisted electrodeposition and differential chemical etching technique. The diameter of the MNPs (*d* = 350 nm) is determined by the anodized aluminum oxide template pore size, while the length (*l* = 75 nm, 200 nm, 500 nm) is controlled by the duration by the pulse durations. An increase in the high potential pulse duration of 2 s to 40 s results in an increased in *l* from 75 nm to 500 nm. (**b**) TEM and SAED image of MNP showing polycrystalline structure. (**c**) EDX shows the composition of MNP measured to be Ni_80_Fe_20_. (**d**) Cell viability of HeLa cells after incubation with MNPs (*d* = 150 nm, 200 nm 350 nm), over a period of 0‒48 hours.

Compositionally modulated NiFe cylindrical nanowires were grown using the anodized aluminum oxide (AAO) template-assisted pulsed electrodeposition technique at room temperature (25 °C) in an electrolyte consisting of NiSO_4_ (0.5 M), FeSO_4_ (0.01 M), and H_3_BO_3_ (0.5 M)^16, 34, 35^. The AAO template was dissolved in NaOH (1 M) solution and the released nanowires were thoroughly rinsed with deionized water to de-alkalize. Subsequently, the Fe-rich regions in the nanowires were etched away in dilute HNO_3_ (0.67%) to form the MNPs^16^. The length (*l*) of MNP is controlled by the duration by the pulse durations, while the diameter (*d*) is determined by the AAO pore sizes. The dimensions of the MNPs were characterized by scanning electron microscopy (SEM), as shown in Fig. 4(a). Transmission electron microscopy (TEM) and selective area diffraction pattern (SAED) indicate a polycrystalline structure, as shown in Fig. 4(b). The MNP composition of Ni_80_Fe_20_ as measured by energy dispersive X-ray (EDX), is easily controlled by altering either the electrodeposition potential or the electrolyte composition^36–38^, as shown in Fig. 4(c).

### Cell culture

HeLa cells were grown in T-75 flask as a monolayer in Dulbecco’s Modified Eagle’s Medium (DMEM) supplemented with high glucose (4.5 g/L), L-glutamine (2 mM), 10% fetal bovine serum (FBS) and 1% penicillin/streptomycin. The cell lines were incubated in a humidified atmosphere of 5% CO_2_ and 95% air at 37 °C. The cell cultures were passaged at ~80% confluence using Trypsin-EDTA to detach the cells from the T-75 flask, and then pelleted by centrifugation at 2000 rpm for 5 min. Next, the supernatant was extracted and the cells pellets were re-suspended in cell culture medium. Countess™ automated cell counter was used to count the HeLa cells in suspension and the cells were seeded at 1 × 10^4^ cells/well in a 96-well microtiter plate. All reagents were purchased from Thermo Scientific™. All experiments were done in quadruplicates.

### *In vitro* cell viability assay

The cell viability was quantified using PrestoBlue® cell viability reagent, which is a resazurin-based solution, by using the reducing power of living cells to quantitatively measure the proliferation of cells. PrestoBlue^®^ reagent was performed according to the manufacturer’s protocol. To obtain the biocompatibility of the MNPs, a concentration of 0.1 mg/ml was incubated with the HeLa cells over a period of 0‒48 hours. Subsequently, the cells were washed and incubated with PrestoBlue^®^ reagent for 2 hours. The cell viability was determined using Synergy^TM^ H4 Hybrid Multi-Mode Microplate Reader and the absorbance readings for each well at 570 nm and 600 nm were recorded. The cell viability was expressed as a percentage relative to the cells unexposed to the MNPs. From Fig. 4(d), the survival rates of HeLa cells exposed to *d* = 150 nm, 200 nm and 350 nm MNPs are 88.1%, 83.9% and 83.3%, respectively, displaying high biocompatibility. After 48 hours, the cell survival percentages drop slightly to 83.4%, 83.2% and 82.5%. These results revealed that the 150-nm-diameter MNPs have a higher survival rate as compared to other MNPs. When the HeLa cells are exposed to the MNPs, some MNPs are internalized to the cells due to endocytosis, and accumulated in the digestive vacuoles. This process causes the cell to overload and leads to induced cell death^39, 40^. The cytotoxicity rates of the MNPs are comparable to other commercially available ferromagnetic nanoparticles used for hyperthermia research with HeLa cells at similar concentrations^41^.

### Cell apoptosis

Before and after the magneto-actuated cell apoptosis treatment, light and fluorescence microscopy were used to analyze the HeLa cell death pathway. The cells were stained with 0.4% Trypan Blue (TB) solution to count the number of dead cells. TB is a cell impermeant stain that enters non-viable cells with a compromised membrane and stains it with an intense blue. As Trypan blue is a cell-impermeant chromophore that can quench internal fluorescent staining in dead cells, a separate group of cells were stained with 20 μg/ml of UltraPure™ Ethidium Bromide (EB) to evaluate the apoptotic changes. EB uptake only by non-viable cells with damaged membrane and emits red fluorescence by intercalation into DNA^23, 24^. The HeLa cells were incubated in DMEM medium supplemented with 10% FBS in a humidified atmosphere of 5% CO2 and 95% air at 37 °C for 30 mins. Light and fluorescence images were captured on a Nikon Eclipse Ti-S inverted microscope equipped with DAPI-FITC-TRITC filter at an excitation maximum of 300 nm and 520 nm and an emission maximum of 600 nm. A minimum of 500 cells were counted in each group to obtain a quantitative measure of cell apoptosis at each frequency between 1‒20 Hz. All reagents were purchased from Thermo Scientific™. All experiments were done in quadruplicates.

### Micromagnetic parameters

The magnetization dynamics in NiFe magnetic nanoparticles, with diameters (*d*) of 150‒350 nm and length (*l*) of 50‒500 nm, were studied by means of Object oriented micromagnetic framework (OOMMF) software^42^. The following material parameters for Ni_80_Fe_20_ were used; saturation magnetization (*M*_*s*_) of 860 × 10^3^ A/m, exchange stiffness constant (*A*_*ex*_) of 1.3 × 10^−11^ J/m, magneto-crystalline anisotropy (*k*) of 0, and Gilbert damping constant (*α*) of 0.01. A unit cell size of 5 × 5 × 5 nm was chosen for all simulations.
